# Brief interventions for problem gambling: A meta-analysis

**DOI:** 10.1371/journal.pone.0214502

**Published:** 2019-04-17

**Authors:** Lena C. Quilty, Jeffrey D. Wardell, Thulasi Thiruchselvam, Matthew T. Keough, Christian S. Hendershot

**Affiliations:** 1 Campbell Family Mental Health Research Institute, Centre for Addiction and Mental Health, Toronto, Canada; 2 Department of Psychiatry, University of Toronto, Toronto, Canada; 3 Institute for Mental Health Policy Research, Centre for Addiction and Mental Health, Toronto, Canada; 4 Department of Psychology, University of Manitoba, Winnipeg, Canada; Monash University, AUSTRALIA

## Abstract

**Background:**

Brief interventions have been increasingly investigated to promote early intervention in gambling problems; an accurate estimate of the impact of these interventions is required to justify their widespread implementation. The goal of the current investigation was to evaluate the efficacy of in-person brief interventions for reducing gambling behaviour and/or problems, by quantifying the aggregate effect size associated with these interventions in the published literature to date.

**Methods:**

Randomized controlled trials including the following design features were identified via systematic review: an adult sample experiencing problems associated with gambling; an in-person individual psychosocial intervention of brief duration (≤3 sessions); a control/comparison group; and an outcome related to gambling behaviour and/or problems.

**Results:**

Five records compared brief interventions to assessment only control; using a random effect model, brief interventions were associated with a small but statistically significant reduction in gambling behaviour across short-term follow-up periods versus assessment only control (*g* = -.19, 95% CI [-.37, -.01]). Aggregate effect sizes for gambling problems and long-term follow-up periods were not statistically significant. Five records compared brief interventions to longer active interventions; there was no significant difference between brief interventions and longer active interventions.

**Conclusions:**

Results supported the efficacy of brief interventions for problem gambling compared to inactive control in the reduction of gambling behaviour; no differences were found across brief versus longer interventions for both gambling behaviour and problems. While these findings must be interpreted in the context of the limited number of studies and small magnitude of the combined effect sizes, the current meta-analysis supports the further investigation of the public health impact of these cost-effective interventions.

## Introduction

Brief interventions have been increasingly investigated to promote early intervention in risky health behaviours. Indeed, Screening, Brief Intervention, and Referral to Treatment (SBIRT) protocols have been increasingly applied to promote the early identification and management of substance misuse [1,2]. Such protocols typically involve universal screening for alcohol or drug use, followed by a brief intervention or referral to specialized services if indicated. In this context, brief interventions are commonly one session in duration and comprise brief advice, motivational enhancement, and goal setting to support behavioural change [3]. SBIRT protocols have been applied to alcohol and drug use within a variety of community and health care settings, such as hospital emergency centres, primary care settings, and trauma care centres. Systematic reviews and meta-analyses provide mixed evidence for their efficacy, although greater evidence exists for their beneficial impacts for alcohol use compared to other substances [4–5]. The potential utility of SBIRT protocols to mitigate the negative consequences associated with behavioural addictions such as problem gambling has yet to receive much empirical attention; however, there has been increasing investigation of the potential public health impact of such an approach in those with problem gambling (e.g., NCT03287583). Empirical support for the therapeutic benefit of brief interventions is necessary to support this form of early intervention in problem gambling.

Similar to alcohol use, the majority of adults engage in gambling behaviour that might be described as minimal or “low risk,” with a more limited proportion engaging in riskier gambling habits and in problem or disordered gambling [6]. Problem gambling refers to gambling that causes harm or disruption to one or more life domains, and thus captures a broad continuum of harms associated with gambling behaviour from mild to severe. Other terms such as compulsive gambling, pathological gambling, and gambling disorder are also frequently used in this literature, and differ primarily in gambling frequency and harm severity. Problem gambling is exhibited by approximately 2–3% of the general population whereas the prevalence of pathological gambling or gambling disorder is estimated to be 1% or below [6–9]. Yet, gambling even five times per year has been associated with a variety of negative consequences including medical and psychiatric health outcomes [7], and those gambling at elevated “risk” are more likely to transition to problem or disordered gambling [8–9]. Recent research has estimated that the gambling behaviour of a problem gambler can negatively impact six or more interpersonal relationships (e.g., family, friends, colleagues) [10], effectively underscoring that the impact of problem gambling extends well beyond the individual gambler (see also [11]).

Specialized problem gambling services are accessed to a limited degree by those in need, suggesting that additional treatment approaches may be of value in mitigating the negative consequences associated with gambling involvement. Research has increasingly highlighted the elevated prevalence of comorbid psychiatric disorders in those with problem gambling [12–14]. Importantly, individuals with problem gambling are more likely to present for treatment for those comorbid difficulties than for gambling itself [15–16], highlighting the potential value of SBIRT protocols to identify and manage gambling problems outside of specialized care clinics. Recent investigations have further suggested that brief interventions are associated with therapeutic benefit in problem gambling, with or without comorbid psychiatric conditions. For example, Petry, Weinstock, Ledgerwood, and Morasco [17] demonstrated that a brief intervention of 10 minutes was associated with greater therapeutic benefit than an assessment only control in adults with problem gambling attending treatment for substance use. Petry, Weinstock, Morasco, and Ledgerwood [18] then replicated this effect in an undergraduate sample as well, although a single session of motivational enhancement had more robust effects on outcomes. More recently, Toneatto [19] did not find any statistically significant differences in clinical outcomes in problem gamblers randomized to receive a single session of psychotherapy versus six sessions of cognitive therapy, behaviour therapy, or motivational therapy. Notably, no inactive control condition was included in this investigation.

In summary, individual studies have provided promising evidence for the efficacy of brief interventions for problem gambling. Yet, studies to date have exhibited numerous study design differences such as comparison group, outcome measures, follow-up assessments, and other important study features. The pooling of research evidence via meta-analysis can integrate this accumulated evidence and yield an aggregate estimate of the effect size associated with brief interventions for problem gambling, and inform stakeholders regarding the therapeutic benefits of an SBIRT approach in managing high risk gambling.

In a seminal systematic review and meta-analysis, Cowlishaw et al. [20] identified 14 studies of psychological treatments for problem gambling. Results supported the efficacy of cognitive behavioural therapy in the reduction of gambling behaviour and problems with a medium to very large effect size, but the durability of these effects and the capacity of other interventions to impact gambling problems were unknown. In a more focused systematic review and meta-analysis of motivational interviewing, Yakovenko et al. [21] found that five studies supported the efficacy of motivational interviewing in reducing gambling behaviour with a small effect size, although the durability of these effects was again uncertain. Notably, although both of these meta-analyses included investigations incorporating interventions of brief duration (i.e., three sessions or fewer), the efficacy of brief interventions and the associated aggregate effect size were not isolated and evaluated separately from longer, more intensive interventions.

Two further reviews have commented more specifically on the potential therapeutic benefit of brief interventions for problem gambling. Swan & Hodgins [22] highlighted the potential of both self-directed and clinician-administered brief interventions for problem gambling in a nuanced narrative review. Most recently, Petry, Ginley, and Rash [23] conducted a systematic review and identified 21 studies of psychosocial treatments for problem gambling; ten of these included one or fewer in-person sessions (i.e., brief interventions, personalized feedback, psychoeducation, workbooks, or a combination). This review was supportive of brief feedback or advice, and also simultaneously considered brief interventions administered in person as well as those delivered via other formats (e.g., online, telephone, workbooks). Notably, SBIRT protocols are most commonly delivered in-person during routine healthcare visits, highlighting the value of considering in-person interventions separately. Furthermore, there are reasons to believe that in-person interventions are more efficacious than self-directed interventions [24]. Most importantly, neither of these reviews undertook to quantify the impact of the brief interventions on gambling behaviour or problems through meta-analytic techniques, which limits their ability to draw conclusions about the statistical significance of the overall effect.

The current investigation sought to extend this important line of research, providing the first meta-analysis of outcomes to in-person brief interventions for problem gambling. Although the promise of brief interventions has been recognized by systematic and narrative reviews in the field to date, it is critical to focus specifically on *in-person* interventions most consistent with SBIRT protocols and to quantify their impact (i.e., to obtain aggregate effect sizes not available in narrative or systematic reviews) both to support the design of future investigations of these interventions in applied research and to justify the implementation of these interventions in applied settings. The objective of the current investigation was to determine the efficacy of brief interventions for reducing adult gambling behaviour and/or associated problems in aggregate, with a focus on brief interventions that are delivered in-person and therefore most likely to be incorporated into a fulsome SBIRT protocol.

## Materials and methods

### Search strategy

Records were identified from the following electronic databases: PubMed, PsycINFO, MEDLINE, and EMBASE, from 1990 to September 1, 2017. Records were also identified from grey literature sources, including resources listed in Grey Matters; the websites of the Canadian Centre on Substance Abuse, Centre for Addiction and Mental Health, and Substance Abuse and Mental Health Services Administration; and gambling databases & E-Libraries including the Gambling Research Exchange Ontario knowledge repository, Gambling Research Database, Gambling Research Australia, Responsible Gambling Council E-Library, Australian Gaming Council E-Library, and New Zealand Problem Gambling Library. Ongoing and completed trials were finally identified by searching registries www.clinicaltrials.gov and www.who.int/ictrp/en/. In addition, records were identified from the reference lists of the two meta-analyses of problem gambling conducted to date [20, 21] and of the records identified during the search process outlined above, as well as from the publications that cited these publications. Experts in the field were also contacted for information about ongoing or unpublished studies. Search terms included: SBIRT or SBI, BI or Brief Intervention, Brief Motivational Intervention, or Motivational Enhancement, in combination with Gambling, Problem Gambling, Pathological Gambling, Gambling Disorder, or Gambling Harm, entered separately. For example, the following search terms was used in PsycINFO, using the Boolean term “or” to explode and map terms related to intervention and gambling, which were combined using the Boolean term “and”: (“SBIRT” OR “SBI” OR “BI” OR “brief intervention” OR “brief motivational intervention” OR “motivational enhancement”) AND (“gambling” OR “problem gambling” OR “pathological gambling” OR “gambling disorder” OR “gambling harm). Filters limited this search to the publication years specified above and to publications in the English language. These search terms were informed by recent meta-analyses of SBIRT protocols and motivational interventions for addictions [25–27].

### Selection criteria and process

Studies were selected according to the following inclusion criteria:

1. Language: English
2. Sample: Adolescents and adults ≥ 16 years of age experiencing gambling problems, as assessed by a validated measure (e.g., Problem Gambling Severity Index [28], South Oaks Gambling Screen [29]) or an a priori defined threshold of problem gambling symptoms or severity or of gambling frequency (e.g., at least 1 diagnostic criterion met, at least $100 in gambling expenditures over the past 3 months).
3. Study Design: Randomized controlled trial
4. Intervention: In-person individual psychosocial interventions of brief duration (≤ three sessions)
5. Comparison/control: An active or inactive comparison or control group
6. Outcomes: Gambling (presence/absence, frequency, severity) and/or associated problems, as assessed both pre- and post-intervention by a validated or purpose-built measure. Gambling problems were broadly defined, including any measure of negative consequences or problems as well as the presence or severity of problem gambling or gambling disorder symptoms (e.g., Structured Clinical Interview for DSM-IV Pathological Gambling [30], Addiction Severity Index [31]). All assessment modalities (e.g., self-report, interview) were permitted.

All settings were considered. Group, telephone, or online interventions were not included.

**Fig 1:**
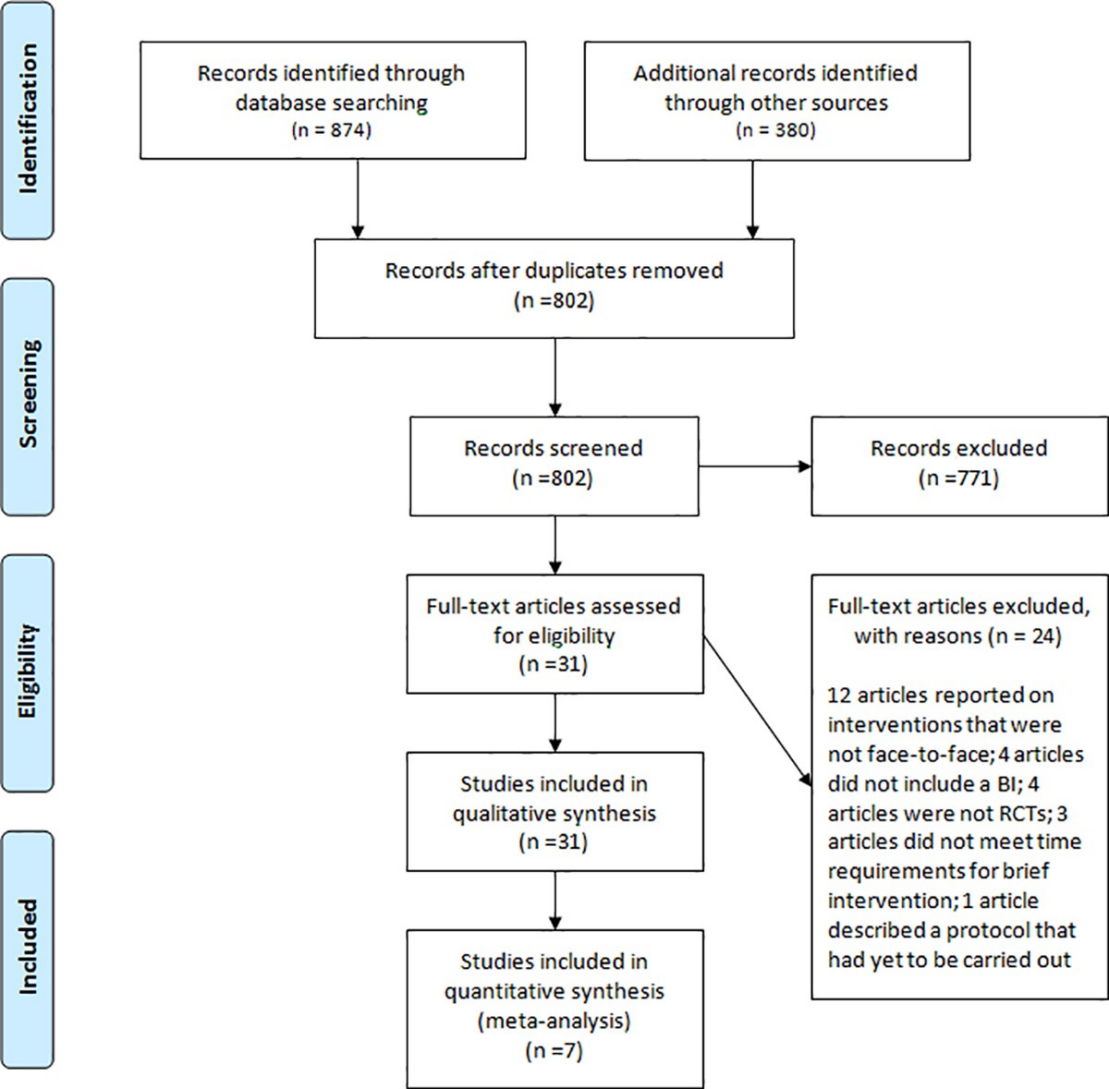
PRISMA flow diagram.

Two research staff independently carried out the following steps: (1) identified all possible records and removed duplicates; (2) screened titles and abstracts of all unique records; and (3) conducted full text reviews for all records not excluded during Step 2, applying a checklist documenting eligibility criteria. A member of the investigator team resolved disagreements and discrepancies (LQ, JW) and another confirmed all records identified for inclusion (MK; Fig 1).

### Data extraction and process

Procedures were consistent with Preferred Reporting Items for Systematic Reviews and Meta-Analyses (PRISMA) criteria [32]. The following data were extracted from records included in analyses: study design features (e.g., setting, clinician), sample features (e.g., size, demographic information, clinical information), screening (e.g., instrument), intervention (e.g., duration, components), outcomes (e.g., instruments, indicators), and bias and fidelity indicators. The following outcomes were extracted at baseline and for each follow-up period (where available): gambling frequency, expenditures, and associated problems. Two research staff independently extracted data to be used in effect size estimates, including sample sizes, means, and standard deviations. A member of the investigator (LQ, JW) team resolved any discrepancies in the values that were extracted by the two research staff.

### Risk of Bias

The Cochrane Risk of Bias Tool was used to evaluate bias at the study level across seven domains: sequence generation; allocation concealment; blinding; incomplete data; selective reporting; conflict of interest; and source of funding [33]. Each domain was given a rating of high, low or unclear risk of bias, according to the guidelines outlined by Hartling et al. [34].

### Statistical analyses

As identified records incorporated qualitatively distinct control conditions, separate meta-analyses were conducted to compare brief interventions to assessment only control conditions, and to longer active comparator conditions. Analyses were conducted with a random effects model in the Comprehensive Meta Analysis (CMA) software package (Comprehensive Meta-analysis, Version 3). The aggregate effect size that was calculated was the bias-corrected standardized mean difference (Hedge’s *g*) between the brief intervention and the comparison conditions on pre-post changes in the gambling outcomes. For records that included multiple brief intervention conditions in the same study, comparisons were combined at the study level taking into account the estimated correlation between the comparisons (consistent with Cochrane review recommendations), which are not independent because the comparisons share a common control group. We used an approach that estimates a sample-size weighted correlation for these comparisons assuming zero correlation between the two independent intervention conditions and a 1.0 correlation between the shared comparison group (see Borenstein et al., [35]). A spreadsheet available for download from the CMA website was used to compute the composite scores (Computing composite scores and variation based on correlation, no version). Thus, each study contributed one composite effect size to the meta-analysis.

Most studies reported multiple gambling outcomes. We conducted separate analyses for each gambling outcome, specifically gambling behaviour (e.g., frequency, dollars spent) versus gambling problem (e.g., South Oaks Gambling Screen, Addiction Severity Index-Gambling scores) outcomes; where multiple indices of gambling behaviour or problem outcomes were available, estimates were collapsed within each study, so each study contributed only one effect size estimate (based on the average effect on gambling behaviour or gambling problems). Also, most records included multiple post intervention time points. We conducted separate analyses for short-term (1–6 month) vs. long-term (7+ month) post-intervention time points. A conservative estimate of .50 was used for pre-post correlation estimates for all pre- vs. post-intervention comparisons. As sample sizes for the study-level effect size estimates were averaged across the sample sizes observed for the follow up time points, sample sizes were adjusted for missing data/participant attrition at follow up. Heterogeneity across studies was evaluated by examining Cochran’s Q statistic and the I^2^ statistic, both of which provide indices of the relative amount of variation in effect sizes that can be attributed to variation across studies versus error variance. Publication bias was evaluated by visually inspecting funnel plots and examining both the Begg and Mazumdar rank correlation test (Kendall’s tau) and Egger’s regression test, all of which represent the association between study size or precision and observed effect size. Absence of bias is supported by a symmetrical funnel plot and nonsignificant values for Kendall’s tau and Egger’s regression.

## Results and discussion

The study selection process is illustrated in Fig 1. We located a total of 874 published and 380 grey literature records. A total of 802 records remained after removing duplicates, and then 31 remained following the screening of titles and abstracts. Records excluded at this stage most commonly did not report original data, did not include an intervention, and did not include critical design features (namely, a randomized controlled trial and a comparison/control group). Of the 31 remaining records, 24 were excluded because they investigated interventions that were not face-to-face (12), that did not include a brief intervention (4), were not a randomized treatment trial (4), did not meet time requirements for brief intervention (3), or were comprised of a protocol only (1). Of the remaining records, seven reports described the results of six randomized comparisons between brief interventions and either an assessment only control condition (five studies) or a longer active intervention (five studies).

### Study characteristics and quality

The sample characteristics of the included studies are described in Table 1, and the intervention content, relevant outcomes, and assessment time points are shown in Table 2. All studies were published from 2008 and after, with sample sizes per treatment condition ranging from 21 to 82. Participants were recruited from academic institutions (2), health care settings (2), and the local community (3), and participant demographics were generally consistent with source of recruitment (e.g., lower mean age in undergraduate samples versus clinical or community samples). All interventions were a single session, ranging in duration from 10 to 90 minutes. Clinicians were generally graduate trainees or research staff. Interventions included a range of components such as personalized feedback, psychoeducation, goal setting, and advice or recommendations. Outcomes included gambling frequency (days per month), expenditures (dollars per month), and associated problems (as assessed by the Problem Gambling Severity Index, South Oaks Gambling Screen, or other validated instruments).

**Table 1:** Features of included randomized controlled trials.

Study details [author(s), year; design; funding]	Recruited sample/setting; Country	Inclusion criteria	Exclusion criteria	Number of randomized participants [treatment arm: n]	Demographics of randomized participants [mean age; sex]
**1. Diskin & Hodgins, 2009 [36]; Funded by the Alberta Gaming Research Institute**	Media-recruited individuals with gambling problems; Canada	**(1)** >17 years of age; **(2)** Scored ≥3 on the PGSI-CPGI; **(3)** Not receiving treatment for problem gambling at time of study; **(4)** Had gambled in the 2 months preceding screen; **(5)** Willingness to provide collateral informant and follow-up data	Did not meet inclusion criteria.	Motivational Interviewing: 42 Control Interview: 39	45 years; 35 f and 46 m
**2. Larimer et al., 2012 [37]; Funded by the National Institute on Mental Health**	Sophomores/ Juniors at large university; United States	**(1)** Scored ≥3 on the SOGS	**(1)** Did not meet eligibility criteria of ≥3 on the SOGS	Personalized Feedback: 52 Cognitive Behavioural Intervention: 44 Assessment Only: 51	21.23 years; 51 f and 96 m
**3. Petry et al., 2008 [17]; Funded by the Patrick and Catherine Weldon Donaghue Medical Research Foundation and the National Institutes of Health**	Individuals at substance abuse treatment clinics and medical clinics; United States	**(1)** ≥ 18 years of age; **(2)** Answered yes to ≥ 3 items on the SOGS; **(3)** ≥ $100 total wagered in the 2 months preceding screen; **(4)** ≥ 4 gambling days in the 2 months preceding screen	**(1)** Reading level < fifth grade; **(2)** suicidal intentions in past month preceding screen; **(3)** psychotic symptoms in past month preceding screen; **(4)** interest in more intensive treatment for gambling than provided in study	Brief Advice: 37 Motivational Enhancement Therapy: 55 Motivational Enhancement Therapy + Cognitive Behavioural Therapy: 40 Assessment Only: 48	43.5 years; 72 f and 108 m
**4. Petry et al., 2009 [18]; Funding NR**	Students at colleges and universities; United States	**(1)** ≥ 18 years of age; **(2)** scored ≥3 on the SOGS; **(3)** ≥$100 total wagered in the 2 months preceding screen; **(4)** ≥4 gambling days in the 2 months preceding screen	**(1)** Psychotic symptoms in past month preceding screen; **(2)** suicidal intentions in past month preceding screen; **(3)** interest in more intensive treatment for gambling than provided in study	Brief Advice: 32 Motivational Enhancement Therapy: 30 Motivational Enhancement Therapy + Cognitive Behavioural Therapy: 21 Assessment Only: 34	20.3 years; 18 f and 99 m
**5. Petry et al., 2016 [38]; Funding NR**	Patients at substance abuse treatment clinics; United States	**(1)** ≥ 18 years of age; **(2)** DSM-IV criteria met for alcohol, cocaine, opioid or marijuana use disorder; **(3)** > 4 gambling days in the 2 months preceding screen; **(4)** > $100 total wagered in the 2 months preceding screen; **(5)** scored > 3 on SOGS based on the 2 months preceding screen	**(1)** Suicidal intentions; **(2)** Active psychotic symptoms at time of screen; **(3)** Inability to read or cognitive impairment; **(4)** receiving treatment for gambling at time of study; **(5)** interest in more intensive treatment for gambling than provided in study	Brief Advice: 66 Motivational Enhancement Therapy + Cognitive Behavioural Therapy: 82 Psychoeducation: 69	41.95 years; 68 f and 149 m
**6a. Toneatto & Gunaratne, 2009 [39]** **b. Toneatto, 2016 [19]** **Funded by Ontario Problem Gambling Research Centre**	Individuals in the Greater Toronto Area community; Canada	**(1)** ≥1 symptom endorsed for pathological gambling as per DSM-IV; **(2)** Active gambling in the past month at the time of screening; **(3)** Not receiving other treatment for problem gambling at the time of study	**(1)** Psychiatric crisis at the time of study, requiring immediate attention; **(2)** Psychosocial crisis at the time of study (i.e., homelessness) requiring immediate attention	Cognitive Therapy: 25 Behaviour Therapy: 24 Motivational Therapy: 22 Minimal Intervention: 28	47.5 years, 27 f and 73 m

SOGS: The South Oaks Gambling Screen; PGSI-CPGI: Problem Gambling Severity Index—Canadian Problem Gambling Index; NR: Not reported

**Table 2:** Interventions and outcomes.

Reference [author(s); year]	Brief intervention(s)	Brief intervention(s) content	Brief intervention(s) therapists	Comparison group: Longer interventions	Comparison group: Control	Follow-up [frequency]	Study Outcomes
**1. Diskin & Hodgins, 2009 [36]**	Motivational Interviewing	Single, manualized session (average duration of ~76 minutes)	2 doctoral students	N/A	Assessment only	3, 6, 9 and 12 months	**(1)** days gambled per month; **(2)** dollars gambled per month; **(3)** gambling problem severity (GSI, PGSI, SOGS)
**2. Larimer et al., 2012 [37]**	Personalized Normative Feedback	Single, 60–90 minutes, individual session, with feedback on patterns, norms, expectancies, consequences, and beliefs related to individual’s gambling.	Largely clinical psychology graduate students trained by study authors	Cognitive Behaviour Intervention (4–6 weekly 1 hour sessions in group format)	Assessment only	6 months	**(1)** gambling frequency based on GQPN; **(2)** gambling expenditure based on GQPN; **(3)** gambling problem severity (GPI, DSM-IV criteria)
**3. Petry et al., 2008 [17]**	1. Brief Advice	Single, 10-minutes session including personalized feedback on gambling, brief recommendations and handout.	9 Bachelors to Masters level therapists	Motivational Enhancement Therapy + Cognitive Behaviour Therapy (1 session of MET + 3 sessions of CBT)	Assessment only	6 weeks and 9 months	**(1)** gambling problem severity (ASI-G); **(2)** dollars gambled per month
	2. Motivational Enhancement Therapy	Single, 50-minutes session, including personalized feedback, discussion, and change plan worksheet.					
**4. Petry et al., 2009 [18]**	1. Brief Advice	Single, 10-15-minutes session including personalized feedback on gambling, brief recommendations and handout.	3 Bachelors to Masters level therapists, 2 clinical psychology doctoral students, and 1 PhD psychologist	Motivational Enhancement Therapy + Cognitive Behaviour Therapy (1 session of MET + 3 sessions of CBT)	Assessment only	6 weeks and 9 months	**(1)** gambling problem severity (ASI-G); **(2)** dollars gambled per month; **(3)** days gambled per month
	2. Motivational Enhancement Therapy	Single, 50-minute session, including personalized feedback, discussion, and change plan worksheet.					
**5. Petry et al., 2016 [38]**	Brief Advice	Single, 10-15-minutes session including personalized feedback on gambling, brief recommendations and handout.	5 Bachelors level to Masters level therapists	Motivational Enhancement Therapy + Cognitive Behaviour Therapy (1 session of MET + 3 sessions of CBT)	Psychoeducation (Single, 10-15- minutes session)	2, 5, 8, 12, 16, 20, and 24 months	**(1)** gambling problem severity (SOGS); **(2)** dollars gambled per month; **(3)** days gambled per month
**6a. Toneatto & Gunaratne, 2009 [39]** **b. Toneatto, 2016 [19]**	Minimal Intervention	Single, 90-minutes session including review of assessment results, handout of interventions, practical advice, and summary booklet.	2 Masters level therapists and 2 doctoral level therapists	Cognitive Therapy (6, 1-hour sessions) Behaviour Therapy (6, 1-hour sessions) Motivational Therapy (6, 1-hour sessions)	N/A	12 months	**(1)** % of days gambled; **(2)** expenditures per gambling day; **(3)** gambling problem severity (DSM-IV criteria)

GSI: Global Severity Index; PGSI: Problem Gambling Severity Index; SOGS: The South Oaks Gambling Screen; GPI: Gambling Problems Index; GQPN: Gambling Quantity and Perceived Norms scale; ASI-G: The Addiction Severity Index-Gambling section

Diskin and Hodgins [36] randomized participants to a brief intervention (single session of motivational interviewing) or to an assessment only control condition, and in participants reporting moderate to severe problem gambling, found that those who received the brief intervention endorsed decreased gambling frequency, expenditure, and associated distress 12 months later compared to control. Larimer et al. [37] randomized participants to a brief intervention (single session of personalized feedback), cognitive behavioural therapy (4–6 weekly sessions), or assessment only control. They found that participants reported at least two gambling disorder symptoms and that both the brief intervention and cognitive behavioural therapy were associated with decreased gambling frequency, consequences and symptoms at 6 month follow up compared to control. Petry et al. [17] randomized participants from medical and substance use clinics to a brief advice (single session), motivational enhancement therapy (single session), combined motivation enhancement and cognitive behavioural therapy (four sessions), or assessment only control. This study thus included two brief interventions, and interestingly, found that brief advice was associated with decreased gambling behaviour at week 6 and with recovery status at week 6 and month 9 compared to assessment only control in participants reporting problem or pathological gambling; no other statistically or clinically significant results were found. Petry et al. [18] randomized undergraduate participants to the same treatment arms and found that all intervention groups were associated with decreases in gambling frequency, expenditures, and problems compared to assessment only control, again in participants reporting problem or pathological gambling; however, the motivational enhancement condition exhibited the most robust therapeutic benefits. Petry et al. [38] randomized participants from substance use clinics reporting problem or pathological gambling to brief advice (single session), combined motivational enhancement and cognitive behavioural therapy (four sessions), or psychoeducation (single session), and found that brief advice was associated with decreased gambling frequency at month 5 compared to psychoeducation and that combined psychotherapy was associated with decreased gambling frequency, expenditure, and problems at month 5 compared to psychoeducation, and greater clinically significant improvements in the short- and long-term. Toneatto and colleagues (both [19] and [39] report the same outcomes) randomized participants to cognitive therapy (six sessions), behavioural therapy (six sessions), motivational therapy (six sessions), or a minimal intervention (single session). All four interventions were associated with similar decreases in gambling frequency, expenditures, and problem severity (i.e., no significant differences between study conditions were found) in participants reporting at least six gambling disorder symptoms.

All studies reported that participants were randomized to study groups, although the randomization method in one study was not described [37]. Potential biases were identified across several domains (see Table 3), where information regarding allocation concealment (four studies; [17, 19, 36, 37]) and blinding of outcomes (four studies; [17, 19, 37, 38]) was unclear or not provided. Further, funding source (two studies; [18, 38]) and lack of conflict of interest (four studies; [17, 19, 36, 38]) was not explicitly confirmed in several studies, and therefore also unclear or not provided. Finally, some studies utilized last observation carried forward or did not report study registration, and were therefore rated as having potential high risk for bias associated with attrition (three studies; [19, 36, 37]) and selective outcome reporting (four studies; [17, 19, 36, 38]).

**Table 3:** Estimated potential risk of Bias.

Study details [author(s), year]	Conflict of Interest	Funding Source	Selective Outcome Reporting	Attrition	Blinding	Allocation Concealment	Randomization
**1. Diskin & Hodgins, 2009 [36]**	Unclear	Low	High	High	Low	Unclear	Low
**2. Larimer et al., 2012 [37]**	Low	Low	Low	High	High	Unclear	Unclear
**3. Petry et al., 2008 [17]**	Unclear	Low	High	Low	High	Unclear	Low
**4. Petry et al., 2009 [18]**	Low	High	Low	Low	Low	Low	Low
**5. Petry et al., 2016 [38]**	Unclear	High	High	Low	High	Low	Low
**6a. Toneatto & Gunaratne, 2009 [39]** **b. Toneatto, 2016 [19]**	Unclear	Low	High	High	High	Unclear	Low

### Brief intervention vs. assessment only control

**Fig 2:**
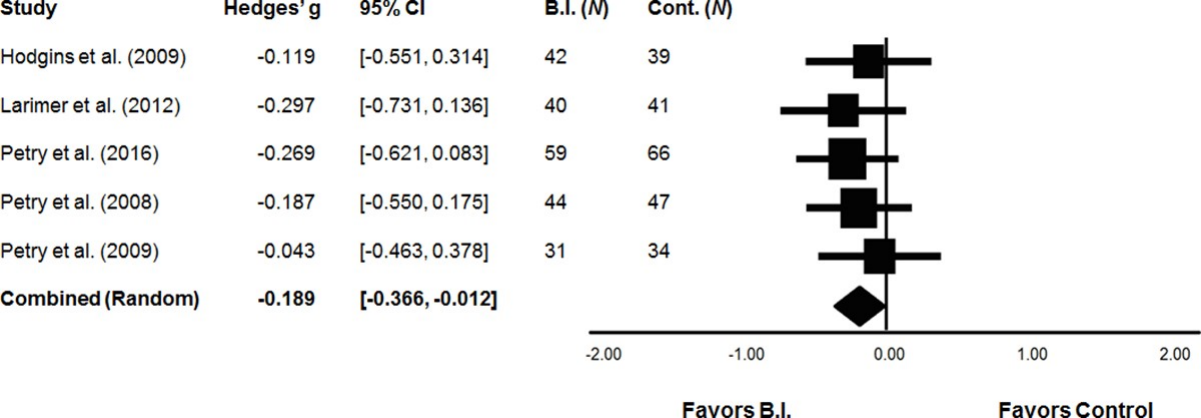
Efficacy of brief interventions vs. assessment only control conditions.

Of the six studies identified, five compared brief interventions to assessment only control conditions. Brief interventions included motivational interviewing/enhancement, personalized feedback, and brief advice. Two studies included two brief intervention groups; these effects were combined at the study level taking into account the correlation among the non-independent comparisons (see Data Analysis). A meta-analysis of short-term gambling behaviour comprised a final analyzed sample of 443 participants across five studies, including 216 receiving a brief intervention and 227 assessment only controls. Using a random effect model, brief interventions were associated with significant reductions in short-term gambling behaviour versus assessment only control (*g* = -0.19, SE = 0.09, 95% CI = -0.37, -0.01; Fig 2). The analysis of long-term gambling behaviour comprised a final analyzed sample of 340 participants across four studies, including 167 receiving a brief intervention and 173 assessment only controls. Effect estimates for long-term changes in gambling behaviour was not statistically significant (i.e., 95% CI contains zero): *g* = -0.17, SE = 0.10, 95% CI [-0.36, 0.04]. Similarly, a meta-analysis of short-term gambling problems comprised a final analyzed sample of 362 participants across four studies, including 174 receiving a brief intervention and 188 assessment only controls. Effect estimates for short-term changes in gambling problems was not significant: *g =* -0.13, SE = 0.12, 95% CI [-0.36, 0.09]. Finally, the analysis of long-term gambling problems comprised a final analyzed sample of 328 participants across four studies, including 164 receiving a brief intervention and 164 assessment only controls. Effect estimates for long-term changes in gambling problems was also not significant: *g =* -0.20, SE = 0.13, 95% CI [-0.46, 0.06] for long-term changes in gambling problems.

There was little evidence for heterogeneity in the effect sizes across studies for the short-term, *Q*(4) = 1.00, p = .909, I^2 =^ 0.00, and long-term, *Q*(3) = 0.72, p = .0.87, I^2 =^ 0.00, gambling behaviour outcomes. Heterogeneity was somewhat greater for the gambling problems outcomes, but still low to moderate overall: *Q*(3) = 4.12, p = .249, I^2 =^ 27.20% for short-term problems, and *Q*(3) = 4.84, p = .184, I^2 =^ 37.99%.

### Brief intervention vs. longer active interventions

Of the six studies identified, five compared brief interventions to longer active interventions. Brief interventions included personalized feedback, brief advice, and provision of supporting materials. Longer active interventions included cognitive therapy, behavioural therapy, motivational therapy, combined cognitive behavioural therapy and combined motivational enhancement and cognitive behavioural therapy. Two studies included two brief intervention conditions, and one study included three longer active comparator conditions; in each case, these conditions were combined at the study level. A meta-analysis of short-term gambling behaviour outcomes comprised a final analyzed sample of 381 participants across five studies, including 201 receiving a brief intervention and 180 active controls. No significant difference between brief interventions and longer active interventions was found (*g* = 0.01, se = 0.09, 95% CI -0.18, 0.20). Similarly, the analysis of long-term gambling behaviour comprised a final analyzed sample of 286 participants across four studies, including 148 receiving a brief intervention and 138 active controls. Again, no significant difference between brief interventions and longer active interventions was found (*g* = 0.04, se = 0.11, 95% CI -0.17, 0.25). A meta-analysis of short-term gambling problems comprised a final analyzed sample of 332 participants across four studies, including 174 receiving a brief intervention and 158 active controls, and located no significant difference between brief interventions and longer active interventions (*g* = 0.11, se = 0.10, 95% CI -0.09, 0.32). Finally, the analysis of long-term gambling problems comprised a final analyzed sample of 286 participants across four studies, including 148 receiving a brief intervention and 138 active controls, and no significant difference between brief interventions and longer active interventions was found (*g* = 0.09, se = 0.11, 95% CI -0.12, 0.30).

There was little evidence for heterogeneity in the effect sizes across studies for short-term gambling behaviour, *Q*(4) = .44, p = .98, I^2 =^ 0.00, and long-term gambling behaviour, *Q*(3) = 0.14, p = 0.99, I^2 =^ 0.00. Heterogeneity was more variable for the gambling problems outcomes: there was little evidence of heterogeneity for long-term gambling problems, *Q*(3) = .80, p = .85, I^2 =^ 0.00, and evidence for a small amount of heterogeneity in short-term gambling problems outcomes, *Q*(3) = 3.06, p = .38, I^2 =^ 2.09%.

### Publication Bias

**Fig 3:**
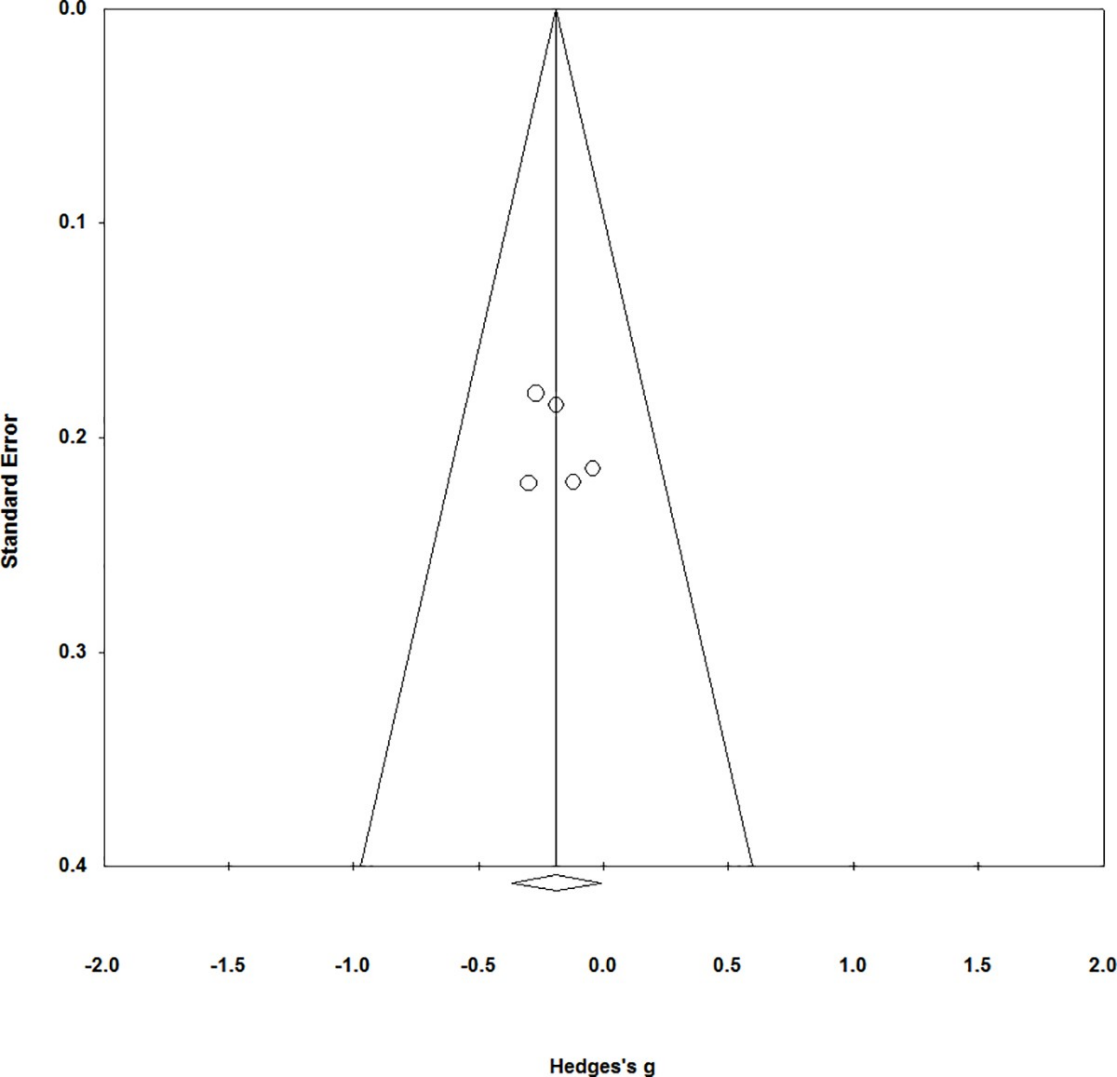
Funnel plot for the meta-analysis of brief interventions vs. assessment only control conditions.

Fig 3 shows the funnel plot for the meta-analysis of brief interventions vs. assessment only for gambling behaviour. As shown, the plot appears to be relatively symmetrical and thus does not suggest the presence of publication bias. The funnel plots associated with the other analyses conducted were similarly symmetric, but are not presented here as their effect size estimates were not statistically significant. Further, Kendall’s tau and Egger’s regression were not statistically significant for any of the meta-analyses conducted (all *ps >* .*05*). However, it is important to note that the small number of studies limits our ability to interpret the funnel plots and results in low statistical power for the publication bias metrics [40]. Thus, although these data do not suggest the presence of publication bias, we are not able to rule out publication bias either.

## Conclusions

Brief intervention within an SBIRT protocol focuses on increasing awareness and knowledge regarding risky health behaviours, as well as working toward increased motivation and capacity for change. The potential public health impact of SBIRT protocols in minimizing the negative consequences associated with gambling involved has been increasingly discussed, and recent narrative and systematic reviews have further highlighted the promise of brief interventions for problem gambling. However, reviews to date have combined in-person, telephone, online, and even self-directed interventions, and importantly, have not calculated an aggregate effect size. The current review doubles the number of investigations of brief interventions included in the seminal meta-analysis of problem gambling treatment [20] as well as a more focused meta-analysis [21], neither of which specifically isolated and evaluated the efficacy of in-person brief interventions. The current investigation located five randomized trials comparing brief interventions for problem gambling to assessment only control conditions, and supported the efficacy of brief interventions compared to assessment only control for gambling behaviours assessed within a shorter time period (six months or less after the intervention). It is notable that the effect sizes associated with individual studies and with this analysis were small in magnitude; indeed, the 95% confidence interval for this meta-analysis is very close to including 0. Nevertheless, this effect size is statistically significant (i.e., the confidence interval suggests that the effect is reliably different from zero), and the small magnitude is in line with previous investigations of brief interventions and reasonable in light of their intensity [5].

The current investigation further located five randomized trials comparing brief interventions to longer active interventions, and did not find differences across single- versus multi-session interventions for both gambling behaviour and problems. It is critical to note, however, that this analysis does not reflect an unbiased comparison of the efficacy of single versus multi-session interventions and should not be interpreted to suggest that brief interventions exhibit therapeutic effects that are equivalent to traditional longer treatments. More specifically, our search strategy identified investigations of brief interventions rather than longer interventions, precluding the ability to generate and compare effect sizes associated with single- and multi-session interventions versus inactive control. Indeed, the effect sizes associated with CBT versus inactive control in a previous meta-analysis [20] were substantively larger than that for brief interventions versus inactive control recovered here, highlighting that these results should not support broad interpretations that single versus multi-session interventions are equivalent in their impact. Finally, publication bias is not possible to fully assess or rule out, based on the limited number of studies conducted.

As a whole, results support the continued evaluation of in-person brief interventions for problem gambling, particularly in additional clinical settings and as part of an SBIRT protocol. In particular, results indicate that brief in-person interventions are associated with a small but significant impact on gambling behaviours at short-term follow-up within at-risk samples. Notably, although these studies included a minimum level of gambling frequency and problems to be eligible for participation, almost all participants endorsed moderate or greater levels of gambling risk, and the majority exhibited problem or pathological gambling. For example, all studies by Petry and colleagues [17, 18, 38] included participants endorsing problem or pathological levels of gambling, and both Diskin and Hodgins [36] and Toneatto and colleagues [19, 39] included participants endorsing clinically significant problem gambling (the former characterizing participant problem gambling as “moderate to severe” and the latter reporting that participants exhibited six to seven diagnostic criteria, with over 80% meeting full criteria). Larimer et al. [37] included participants with the lowest level of gambling problems, with participants endorsing approximately two diagnostic criteria, and about 10% meeting full criteria. Thus, results supporting the value of brief interventions for short-term gambling compared to assessment control were primarily based on participants exhibiting moderate risk or greater, rather than lower risk. These brief interventions may be usefully administered earlier in the progression of the illness to prevent the escalation of problem gambling to a fulsome gambling disorder. From a methodological perspective, these results suggest that it may be circumspect to avoid the use of brief intervention as a control condition in treatment trials, as its modest but reliable therapeutic benefits may reduce power to detect effects of investigational treatments. This investigation is a meaningful extension of previous meta-analyses [20–21], as it includes several trials that have been published since their completion, the consideration of multiple modalities, and the specific evaluation of the aggregate effect size and publication bias associated with brief in-person interventions for problem gambling, critical to justify continued investigation in applied settings. Nevertheless, this investigation further highlights the limited number of published investigations of brief interventions in the literature; our results therefore represent a call to action for additional trials across a range of research groups, clinical settings, and clinician types to resolve this notable gap in the literature.

Brief interventions are variably defined and administered. Consistent with Babor [41], we had a cut-off of three sessions but found that all identified records in fact included brief interventions a single session in duration. Recent reviews of brief interventions for substance misuse have similarly found that most studies utilized brief interventions which consisted of a single session [5, 42]. Yet, these and other reviews have utilized or advocated for a longer duration cut off for defining brief interventions [43], which would result in a different characterization of the interventions included within the current investigation (i.e., some longer active controls would be described as a brief intervention according to this definition). Previous research has usefully highlighted the distinction between brief interventions indicated by opportunistic screening in primary care settings compared to those more commonly found in specialized services, which differ in length, structure, theoretical foundation, and other features [44]. As noted in this seminal review, the former are frequently supported by comparisons to inactive controls whereas the latter are often not found to be different from longer active interventions. Notably, individual studies are rarely fully powered to permit non-inferiority analyses and interpretations. Future research sufficiently powered to quantify the efficacy of “very brief” versus “extended brief” interventions for problem gambling would be of substantial value (see [45]).

Future research may further usefully consider the public health impact of other intervention modalities (e.g., telephone administration), including those that do not require clinician involvement at all (e.g., online supports). Telephone support is a common referral for those exhibiting risky gambling worldwide, and evidence does support their capacity to impact gambling behaviours and problems [46]. A recent systematic review suggested that evidence for the efficacy of computerized brief interventions for problem gambling is modest; however, these have yet to be quantified [23]. Component analyses would be a useful extension of this research as well, as brief interventions, extended brief interventions, and longer protocols comprise numerous therapeutic elements in common, which is a challenge to the identification of both the nature and the intensity of interventions likely to have the greatest impact. Future research would benefit from the incorporation of health economic analyses as well, to support the public health impact of these low intensity but highly accessible forms of support.

The current investigation provides useful evidence for the promise of brief interventions in the treatment of problem gambling; however, these results must be considered in light of several study limitations. First, only a limited number of studies were identified, which limited statistical power and precluded the investigation of numerous moderators of clinical outcomes. Furthermore, these limited studies were conducted by four independent research teams, which may have limited the variability in research design and intervention protocol and contributed to biased effect size estimates and lower generalizability of results. Although research suggests that reviews incorporating few manuscripts may yield robust evidence and conclusions [47], additional trials are clearly required to bolster these effects. Second, some studies contrasting brief interventions to longer active conditions included fewer than 25 participants per treatment condition, which is a minimum recommendation followed by some recent meta-analyses in the field [20], but not others [21]. In the absence for a strong consensus regarding the minimum number of participants per condition, however, the current investigation erred on the side of over-inclusiveness. It is notable that the effect sizes from studies including fewer than 25 participants per treatment condition are weighted according to sample size in the meta-analysis. Third, although the current investigation was not restricted by geographical region, all studies identified were conducted in North America, which may impact the generalizability of our results to other geographical regions. Fourth, interrater reliability for study screening and inclusion is not available, and would valuable in future investigations.

Research has accrued for the efficacy of brief interventions in the treatment of addictive behaviours [25]. In line with this growing foundation of evidence, the current investigation provides some support for the efficacy of brief interventions in the treatment of problem or disordered gambling. These interventions therefore require limited commitment of resources for both clinicians and patients, and may be feasibly incorporated into the regular consultation period of many front-line service providers. Results provided evidence to support a significant benefit of brief interventions in the reduction of gambling involvement and associated problems, but must be interpreted with caution in light of the limited number of studies conducted to date. Studies in primary care and community centres would usefully extend this line of research, and provide invaluable evidence for the practicability of this approach in real-world settings [48].

## Supporting information

S1 File PRISMA checklist. (DOC)
